# The Prevalence of Asthma and Declared Asthma in Poland on the Basis of ECAP Survey Using Correspondence Analysis

**DOI:** 10.1155/2013/597845

**Published:** 2013-01-16

**Authors:** M. Zalewska, K. Furmańczyk, S. Jaworski, W. Niemiro, B. Samoliński

**Affiliations:** ^1^Department of Environmental Hazards Prevention and Allergology, Medical University of Warsaw, Warsaw, Poland; ^2^Department of Applied Mathematics, Warsaw University of Life Sciences, Warsaw, Poland; ^3^Department of Econometrics and Statistics, Warsaw University of Life Sciences, Warsaw, Poland; ^4^Faculty of Mathematics, Informatics and Mechanics, University of Warsaw, Warsaw, Poland; ^5^Faculty of Mathematics and Computer Science, Nicolaus Copernicus University, Toruń, Poland

## Abstract

Results of epidemiological and public health surveys are often presented in the form of cross-classification tables. It is sometimes difficult to analyze data described in this way and to understand relations between variables. Graphical methods such as correspondence analysis are more convenient and useful. Our paper describes an application of correspondence analysis to epidemiological research. We apply the basic concepts of correspondence analysis like profiles, chi-square distance to medical data concerning prevalence of asthma. We aim at describing the relationship between asthma, region, and age. The data presented in this paper come from Epidemiology of Allergy in Poland (ECAP) survey in years 2006–2008. Correspondence analysis shows that there is a fundamental difference in the structure of age groups for people with symptoms compared to those who have declared asthma (regardless of the level of symptoms of asthma and the level of declaration). The variable which best differentiates declared asthma in all regions is “wheezing and whistling.” Correspondence analysis also shows significant differences between locations. Our analyses are performed in the R package “ca”.

## 1. Purpose

**Figure 1:**
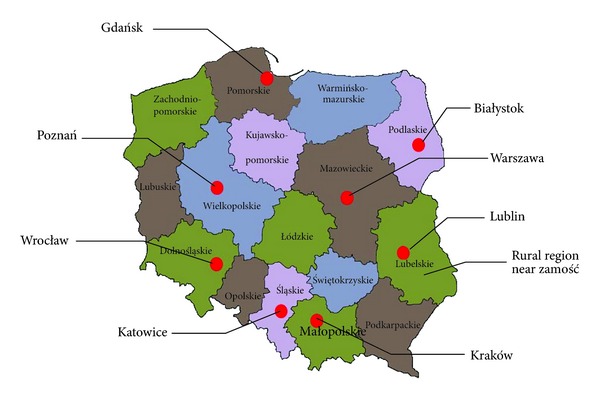
Map of Poland. Red points indicate the major cities in ECAP study, Katowice: Śląskie district, Zamość: a rural region in Lubelskie district, Kraków: Małopolskie district, Wrocław: Dolnośląskie district, Lublin: Lubelskie district, Gdańsk: Pomorskie district, Warszawa: Mazowieckie district, Poznań: Wielkopolskie district, Białystok: Podlaskie district) Source: ECAP report [1].

The analysis is based on data from the ECAP survey [1]. ECAP is a questionnaire-based survey on International Study of Asthma and Allergies in Childhood (ISAAC [2]) and European Community Respiratory Health Survey (ECRHS [3]). In our analysis we consider 18617 subjects (50.4% adults aged 20–44 years, 24.2% children 6-7 years, and 25.4% children aged 13-14 years, 53.8% female and 46.2% male). The structure of symptoms of asthma and the structure of declared asthma are studied. Both structures are related to three age groups: children aged from 6 up to 7 years (Ch1), children aged from 13 to 14 years (Ch2), and adults aged from 20 to 44 years (Ad). The study examines differences and similarities of these structures in eight major Polish cities: Warszawa, Lublin, Białystok, Gdańsk, Poznań, Wrocław, Katowice, Kraków, and in one rural area near Zamość. The locations are presented in Figure 1. 

We have taken into account the following two symptoms: “whistling and wheezing in breathing” and “difficulty in breathing.” First of these symptoms is known to be a good indicator of asthma [4]. The symptoms concern the years that preceded the moment of survey. “Declared asthma” is understood as a disease which the respondent reported in the response to a question of the interviewer. We consider the problem of undetected asthma in different regions and different age groups of patients.

## 2. Statistical Methods

Correspondence analysis [5] now becomes an important tool in epidemiological research [6–9]. It is useful in analyzing multivariate data, most often given in a cross-tab form (cross-classification contingency tables). Traditional approach to such data is to use chi-square tests and, in the special case of 2 × 2 tables, standard epidemiological measures odds ratio (OR) and relative risk (RR). However, this approach is not adequate if we are to discover and explain associations between many variables (features and symptoms). Chi-square test can only tell us that there are statistically significant dependencies. More sophisticated methods are needed to identify the form, direction, and strength of these dependencies. Correspondence analysis with its graphical output allows to describe and easily interpret the structure of such data. Strong association between variables is clearly shown as closeness of the corresponding points in a graph.

Our paper uses correspondence analysis applied to the relative frequency of cases (see Tables 1, 3, 5, 7, 9, and 11) instead of absolute counts. This method has been chosen because the sample sizes in individual cities and age groups significantly differ from one another. Thus in our paper we use correspondence analysis in a nonstandard way.

Let us explain the criterion we are going to use for comparisons. First, we try to determine how to compare the structure of declared asthma on the one hand and the structure of symptoms on the other hand, in different locations and different age groups. The method we use allows us to better understand these two structures and their mutual relation. In our paper, the emphasis is on the relative ratio of frequency of examined features in the three age groups. Thus, we are less interested in the levels of incidence of symptoms and declared asthma in each age group. These levels depend on many factors which we cannot fully identify. Factors affecting the frequency of the analyzed features may also influence the level and type of pollutants in the air, in water, and in food products. They also may influence the awareness of the respondents as to which symptoms can be regarded as typical, and are associated with different levels of diagnosis of allergic diseases by physicians. For example, if the levels of declared asthma in two regions are different, this does not necessarily mean that the prevalence of asthma varies significantly in these regions. Just one of these regions may have less well-developed prevention.

Therefore, the structure we are trying to understand and describe is the distribution of the percentage of people with properties of interest to us: declared asthma and having symptoms of asthma, assuming that the three age groups are equinumerous. Let us explain this using Tables 1 and 2 as an example.  Table 1 shows that percentages of respondents having symptoms (wheezing and whistling) in different locations (Katowice, Zamość, Kraków, Wrocław, Lublin, Gdańsk, Warszawa, Poznań, and Białystok) and the age groups (Ch1, Ch2, Ad). For example in Katowice the percentage of respondents was, respectively, 19%, 10%, and 12%. In contrast, Table 2 shows the proportion of respondents having symptoms in the three age groups. For example in Katowice, 46%, 24%, and 29% of people with “wheezing and whistling” belong to the groups younger children (Ch1), older children (Ch2), and adults (Ad), respectively, (under the assumptions that the groups are equinumerous). In other words, we adopt the Bayesian philosophy and try to estimate the posterior distribution of the age groups given occurrence of symptoms, under the uniform prior distribution.

Let us explain the advantages of the above described approach, using the following hypothetical example. Imagine that the surveyed group has 1,000 people and the number of people with symptoms of asthma in each age group equals, respectively, 3, 6, and 1 or, alternatively, 300, 600, and 100. Although the incidence in individual cases differ dramatically (3/1000, 6/1000, 1/1000 or 300/1000, 600/1000, 100/1000) the structure in both cases has the same form (30%, 60%, and 10%). The assumption that the groups are equinumerous is somewhat arbitrary, but it is needed because the age structure of the various Polish regions is not identical. In the language of correspondence analysis, the structure under examination will be called a profile.

In the following five sections we will discuss five problems of medical relevance. The first problem will be described in a more detailed way to introduce some general ideas and notations.

## 3. Comparison of “Wheezing and Whistling” with Declared Asthma

We recall that our study concerns data from the ECAP survey. In this section we examine two variables: “wheezing and whistling,” a symptom of asthma and declared asthma. The three age groups (Ch1, Ch2, and Ad) and nine locations are the same as described inSection 1. 

The meaning of symbols In Tables 1 and 2 is the following: Ch1; children aged 6-7 years, Ch2; children aged 13-14 years, Ad; adults aged 20–44 years. Warszawa (Wa), Lublin (L), Białystok (B), Gdańsk (Gd), Poznań (Poz), Wrocław (Wr), Katowice (Kat), Kraków (Kr), rural region in the area of Zamość (Zam). Symbol “o” after the abbreviated name of a city/region stands for “symptom,” symbol “a,” analogously stands for “declared asthma.” This notation will be used also in the rest of our paper.

**Figure 2:**
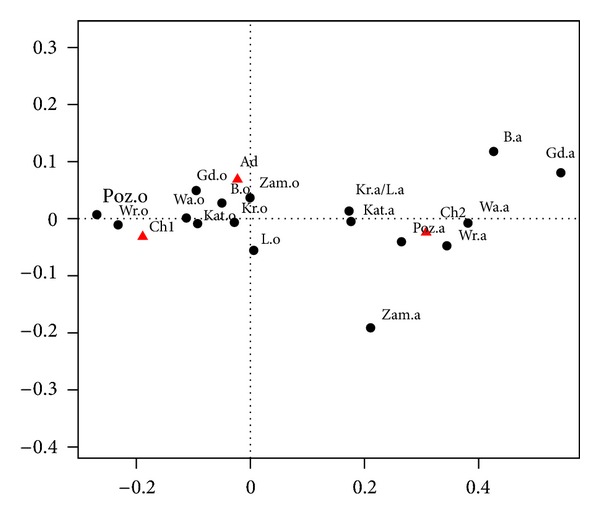
Correspondence map for “wheezing and whistling” and declared asthma.

To compare the relative frequencies in different cities we use correspondence analysis. The essence of this method is its graphic form. Figure 2 displays an output of correspondence analysis. Rows and columns of cross-classification table are represented as points. In Table 2, rows correspond to cities and columns—to age groups. Black dots in Figure 2 represent the structure of wheezing and whistling (symbol “o” after the abbreviated name of a city) and declared asthma (symbol “a,” analogously). For example, “Poz.o” stands for “Poznań; respondents with wheezing and whistling,” “Poz.a” stands for “Poznań; respondents with declared asthma.” Red triangles represent three different age groups.

More precisely, in Figure 2 we present the relative frequency of profiles (the rows in Table 2). Distances between points in the graph (black dots) are equal to the chi-squared distances between profiles. For example, the distance between the profile for “Kat.o” (Katowice; symptom “wheezing and whistling”) and the profile “War.a” (Warszawa; declared asthma) is

(1) $$\begin{array}{c} \sqrt{\frac{{\left(0.46-0.28\right)}^{2}}{0.39}+\frac{{\left(0.24-0.44\right)}^{2}}{0.32}+\frac{{\left(0.29-0.28\right)}^{2}}{0.29}}. \end{array}$$

Note that the reference point is the average profile (39%, 32%, and 29%). The position of points representing the cities (dots) in relation to points representing the age groups (red triangles) indicates the contribution of the age groups to the profile. The sizes (areas) of dots are proportional to sums of rows in Table 1.

In correspondence analysis, explanatory strength of variables is conveniently described by partitioning of the so-called inertia (variance of the data). The percentage of total inertia explained by the two axes in Figure 2 is 100%. It is not surprising because the row profiles lie on a two-dimensional simplex. The horizontal axis captures 95.5% of inertia, and the vertical axis 4.5%.

### 3.1. Preliminary Conclusions

Let us explain the interpretation of results shown in Figure 2 from the epidemiological point of view. The projection on the first (horizontal) axis clearly shows that in the group of declared asthma there is far greater percentage of younger children (Ch1) than older children (Ch2), and this is regardless of the city although the biggest disparity is visible for Gdańsk, Białystok, Warszawa and Wrocław, and the smallest for Kraków, Lublin, Katowice, and Zamość. The projection on the second (vertical) axis well separates the adult respondents from children (both Ch1 and Ch2). In Zamość there is a relatively small proportion of adults in the group of declared asthma. Let us note that “small” or “great” is understood in relation to the average profile (i.e., 39%, 32%, and 29%, see Table 2) and, consequently, concerns the relative comparison of the cities. For example, in relation to asthma symptoms, the distributions in Zamość, Kraków, Lublin, and Białystok are similar to the average profile, and distributions in Poznań and Wrocław deviate from it. Recall that the sizes of dots in Figure 2 are meaningful: they are proportional to sums of corresponding rows in Table 1. It is clear that declared asthma is not as common as its symptoms. Diagnostics of asthma in both groups of children is significantly different.

We can see that in Figure 2 the black points form two clearly visible clusters. The first cluster, on the left hand side, corresponds to the “wheezing and whistling” variable in different locations, and it is clearly associated with two age groups: “6-7 years” and “Adults” (depicted as red triangles). The cluster on the right hand side corresponds to “declared asthma” and is associated with the age group “13-14 years.” In a group with symptoms of asthma, it appears that there is a higher percentage of younger children and adults than of older children. These proportions are reversed in the group with declared asthma. This phenomenon may be due to two reasons. First, in the group of younger children it is harder to detect asthma, than for older children. Second, older children may not have symptoms, which disappear with age, partly because they are diagnosed and are treated. The largest percentage of asthma symptoms in the group of younger children is in Poznań and Wrocław, and then in Gdańsk, Warszawa, and Katowice. The smallest (around 42%) is in Zamość, Kraków, Lublin, and Białystok.

Correspondence analysis showed an essential difference in the structure of age groups for respondents with symptoms of asthma compared to those with the declared asthma (regardless of the level of symptoms and the level of declaration). It has also demonstrated the difference between the cities. The following cities seem to be outliers from the rest: Poznań and Wrocław (in a group with symptoms) and Gdańsk, Białystok, and Zamość (in the group with asthma declared), see Figure 2.

## 4. Comparison of Breathing Difficulties and Declared Asthma

**Figure 3:**
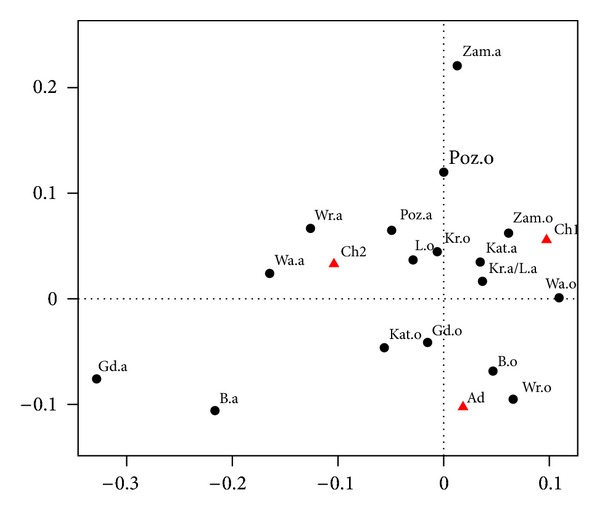
Correspondence map for breathing problems and asthma declared.

The purpose of this chapter is to compare the structure of declared asthma related to breathing problems, a symptom of asthma. We want to show the relationship of specific symptoms (difficulty in breathing) in relation to declared asthma. It will be shown in Tables 3 and 4 and in Figure 3. As before, we use the following symbols: o-symptoms, a-declared asthma.

In Tables 3 and 4 and in Figure 3 we use the same symbols as in Tables 1 and 2 and Figure 2. The horizontal axis captures 61.2% of inertia, and the vertical axis 38.8%.

### 4.1. Preliminary Conclusions

We see that breathing problems occur more frequently in Wrocław and Białystok in adults (Ad) than in children Ch1, Ch2 (see Figure 3). In Zamość, more respiratory problems in children occur in the group Ch1, and less among adults. Another exception is Poznań, where respiratory problems are much more common for both groups of children (Ch1 and Ch2) than for adults (Ad). Surprisingly, in Gdańsk and Białystok, occurrence of respiratory problems among adults is relatively high, while occurrence of declared of asthma is relatively low. We can offer two explanations of this fact. It might be possible that in these cities there is a low detection rate of asthma in adults. Or maybe it is connected with the occurrence of other diseases associated with difficulties in breathing. It is clear that breathing difficulties are not strongly correlated with declared asthma. It may be related to different diseases.

## 5. The Prevalence of Wheezing and Whistling and Breathing Difficulties

**Figure 4:**
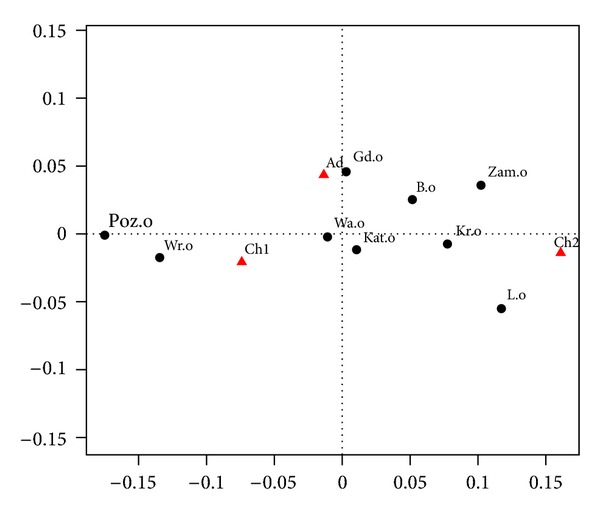
Correspondence map for the wheezing and whistling breathing.

**Figure 5:**
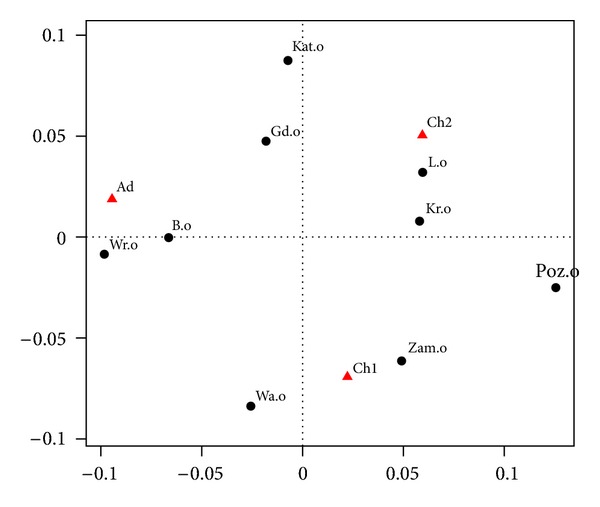
Correspondence map for breathing difficulties.

Now we examine the prevalence of each of the two symptoms “wheezing and whistling” and “breathing difficulties” separately. The results concerning “wheezing and whistling” are presented in Tables 5 and 6 and Figure 4, and the results concerning “breathing difficulties” in Tables 7 and 8 and Figure 5.

### 5.1. Wheezing and Whistling

In Tables 5 and 6 and in Figure 4 we again use the same symbols as in Tables 1 and 2 and Figure 2.

The horizontal axis in Figure 4 captures 91.5% of inertia, and the vertical axis 8.5%.

### 5.2. Preliminary Conclusions

In the group of younger children (Ch1) “whistling and wheezing” occurs most frequently in Wrocław and Poznań, while in the group of adults—in Gdansk, and in the group of older children (Ch2)—in Lublin, Kraków, and Zamość. A level similar to the average profile (46%, 24%, and 30%) is in Warszawa and Katowice for all age groups.

### 5.3. Breathing Difficulties

In Figure 5, the horizontal axis captures 61.0% of inertia, and the vertical axis 39.0%.

### 5.4. Preliminary Conclusions

Among adults problems with breathing occur most frequently in Wrocław and Białystok and to a lesser extent in Katowice and Gdańsk. In the group of older children (Ch2)—in Lublin, Kraków, Poznań, and Katowice, and for younger children in the group (Ch1)—in Zamość and Warszawa.

Moreover, in Wrocław, and Białystok far more adult people (Ad) have breathing problems than in both groups of children (Ch1 and Ch2). In Poznań, Lublin, Kraków, and Zamość more people have breathing problems in groups Ch1 and Ch2 than in adults.

## 6. Declared Asthma

**Figure 6:**
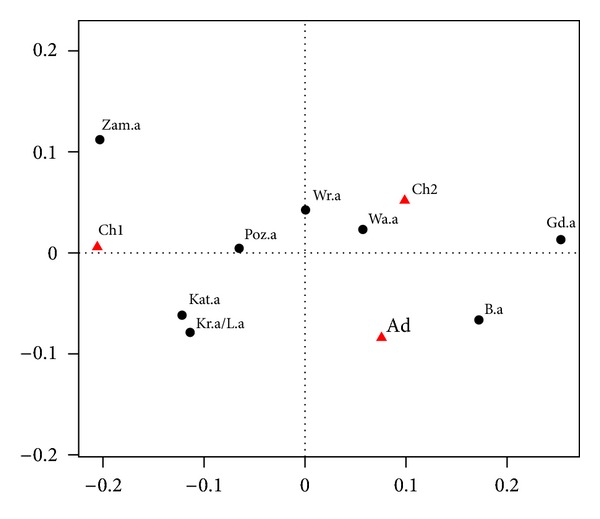
Correspondence map for declared asthma.

We examine the prevalence of declared asthma separately. The results are presented in Tables 9 and 10 and Figure 6.

The horizontal axis in Figure 6 captures 85.6% of inertia, and the vertical axis 14.4%.

### 6.1. Preliminary Conclusions

For younger children in the group Ch1 most cases of asthma were recorded in Zamość, Poznań, Katowice, Kraków, and Lublin. In these cities, asthma occurs significantly more frequently in the Ch1 group than in both Ch2 and Ad groups. Among older children (Ch2), most cases of asthma were reported in Warszawa and Wrocław. The largest group of asthma cases among adult is reported in Białystok.

## 7. Problem of Undetected Asthma

**Figure 7:**
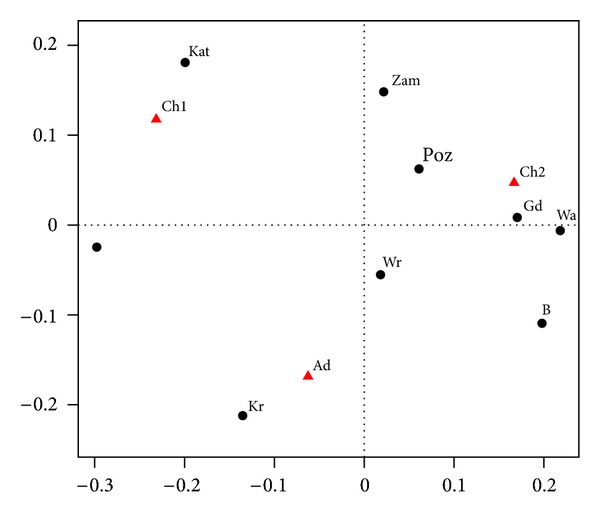
Correspondence map for undetected asthma.

To examine this problem we will consider the pair of variables: declared asthma and “wheezing and whistling” in a different way than in our previous analysis. We regard “wheezing and whistling” as a good indicator of occurrence of asthma. Therefore we are interested in the incidence of declared asthma only among respondents with “wheezing and whistling.” The results are presented in Tables 11 and 12 and Figure 7.

The horizontal axis in Figure 7 captures 67.7% of inertia, and the vertical axis 32.3%.

### 7.1. Preliminary Conclusions

Among younger children (Ch1) the best diagnostics of asthma is in Katowice and the worst is in Białystok, because in Katowice we have the highest percentage (in the group Ch1) of declared asthma among respondents with “wheezing and whistling,” while the lowest percentage is in Białystok. Analogously, among older children (Ch2) the highest percentage is in Gdańsk and Warszawa, and the relatively low in Lublin. Among adults (Ad) the highest percentage is in Kraków, and the lowest in Katowice, Lublin, and Białystok (points corresponding to these cities are located far from the point “Ad” on the graph).

## 8. General Conclusions

It is common knowledge that asthma represents a serious public health problem. According to WHO 235 million people suffer from asthma, among them 30 million in Europe. In some countries up to 20% of population suffer from it. Over 255 thousand of people in the world yearly die of asthma. In Europe asthma is one of the most common chronic noncommunicable diseases in children with average prevalence 5–20%. European Union spends near 17.7 billion EUR per year due to asthma. The overall cost of treating respiratory diseases in Europe is 100 billion EUR annually and is still rising. A better understanding of factors affecting prevalence of asthma is of great importance for finding better strategies for its prevention and treatment.

The research presented here is concerned with asthma problems in Poland [1] which is an important public health issue in our country. However, our conclusions are probably also relevant to other countries. Correspondence analysis shows an essential difference in the structure of age groups for respondents with symptoms of asthma compared to those with the declared asthma (regardless of the level of symptoms and the level of declaration). “Wheezing and whistling” better differentiates declared asthma than “difficulties in breathing.” Our analysis also shows significant differences between age groups and cities. We also consider the problem of underdiagnosed asthma. The map of correspondence analysis indicates locations and age group where this problem may be serious. Declared asthma and its symptoms are more frequent in urban areas than in rural areas. The big difference between prevalence of symptoms of asthma and declared asthma, revealed by our analysis, may suggest directing a prevention program at the improvement of asthma diagnostics in the group in younger children and selected regions. The available funds can be better allocated in this way.

The future research can use correspondence analysis to examine the relation between asthma and such factors as allergic rhinitis, positive skin prick tests, atopic dermatitis, and family history of allergy.

Our results confirm that the graphical output of correspondence analysis is a convenient and flexible tool of detecting interdependencies in big data sets. We can recommend wider use of this method for epidemiological applications.

We propose a simple tool for discovering nonuniform occurrence of symptoms asthma as well as declared asthma in different age groups and different locations. Outlying locations for particular age groups may be therefore given more attention and more careful prevention programs.

The novelty of our approach is in applying the correspondence analysis to the relative frequency instead of absolute counts. This approach has proved useful in presented medical applications.

## Figures and Tables

**Table 1:** Percentages of respondents.

	Age 6-7 (Ch1)	Age 13-14 (Ch2)	Age 20–44 (Ad)
Symptom “wheezing and whistling” “.o”			
Katowice (Kat.o)	19%	10%	12%
Zamość (Zam.o)	12%	8%	9%
Kraków (Kr.o)	21%	13%	14%
Wrocław (Wr.o)	28%	10%	16%
Lublin (L.o)	18%	12%	11%
Gdańsk (Gd.o)	21%	11%	15%
Warszawa (Wa.o)	22%	11%	14%
Poznań (Poz.o)	19%	6%	11%
Białystok (B.o)	17%	10%	12%
Declared asthma “.a”.			
Katowice (Kat.a)	5%	5%	4%
Zamość (Zam.a)	4%	4%	2%
Kraków/Lublin (Kr.a/L.a)	6%	6%	5%
Wrocław (Wr.a)	7%	10%	6%
Gdańsk (Gd.a)	3%	8%	5%
Warszawa (Wa.a)	5%	8%	5%
Poznań (Poz.a)	5%	6%	4%
Białystok (B.a)	2%	4%	3%

**Table 2:** Profiles.

	Age 6-7 (Ch1)	Age 13-14 (Ch2)	Age 20–44 (Ad)
Symptom “wheezing and whistling”			
Katowice (Kat.o)	46%	24%	29%
Zamość (Zam.o)	41%	28%	31%
Kraków (Kr.o)	44%	27%	29%
Wrocław (Wr.o)	52%	19%	30%
Lublin (L.o)	44%	29%	27%
Gdańsk (Gd.o)	45%	23%	32%
Warszawa (Wa.o)	47%	23%	30%
Poznań (Poz.o)	53%	17%	31%
Białystok (B.o)	44%	26%	31%
Declared asthma			
Katowice (Kat.a)	36%	36%	29%
Zamość (Zam.a)	40%	40%	20%
Kraków/Lublin (Kr.a/L.a)	35%	35%	29%
Wrocław (Wr.a)	30%	43%	26%
Gdańsk (Gd.a)	19%	50%	31%
Warszawa (Wa.a)	28%	44%	28%
Poznań (Poz.a)	33%	40%	27%
Białystok (B.a)	22%	44%	33%

Average (average profile)	39%	32%	29%

**Table 3:** Frequency of breathing difficulties and declared asthma.

	Age 6-7 (Ch1)	Age 13-14 (Ch2)	Age 20–44 (Ad)
Symptom breathing problems			
Kat.o	18%	23%	19%
Zam.o	15%	14%	11%
Kr.o	21%	23%	17%
Wr.o	25%	24%	26%
L.o	19%	22%	16%
Gd.o	26%	30%	26%
Wa.o	32%	27%	26%
Poz.o	18%	19%	12%
B.o	18%	18%	18%
Declared asthma			
Kat.a	5%	5%	4%
Zam.a	4%	4%	2%
Kr.a/L.a	6%	6%	5%
Wr.a	7%	10%	6%
Gd.a	3%	8%	5%
Wa.a	5%	8%	5%
Poz.a	5%	6%	4%
B.a	2%	4%	3%

**Table 4:** Row profiles.

	Age 6-7 (Ch1)	Age 13-14 (Ch2)	Age 20–44 (Ad)
Symptom breathing problems			
Kat.o	30%	38%	32%
Zam.o	38%	35%	28%
Kr.o	34%	38%	28%
Wr.o	33%	32%	35%
L.o	33%	39%	28%
Gd.o	32%	37%	32%
Wa.o	38%	32%	31%
Poz.o	37%	39%	24%
B.o	33%	33%	33%
Declared asthma			
Kat.a	36%	36%	29%
Zam.a	40%	40%	20%
Kr.a/L.a	35%	35%	29%
Wr.a	30%	43%	26%
Gd.a	19%	50%	31%
Wa.a	28%	44%	28%
Poz.a	33%	40%	27%
B.a	22%	44%	33%

Average profile	32%	39%	29%

**Table 5:** Frequency of wheezing and whistling breathing.

Symptom wheezing and whistling	Age 6-7 (Ch1)	Age 13-14 (Ch2)	Age 20–44 (Ad)
Kat.o	19%	10%	12%
Zam.o	12%	8%	9%
Kr.o	21%	13%	14%
Wr.o	28%	10%	16%
L.o	18%	12%	11%
Gd.o	21%	11%	15%
Wa.o	22%	11%	14%
Poz.o	19%	6%	11%
B.o	17%	10%	12%

**Table 6:** Row profiles.

Symptom wheezing and whistling	Age 6-7 (Ch1)	Age 13-14 (Ch2)	Age 20–44 (Ad)
Kat.o	46%	24%	29%
Zam.o	41%	28%	31%
Kr.o	44%	27%	29%
Wr.o	52%	19%	30%
L.o	44%	29%	27%
Gd.o	45%	23%	32%
Wa.o	47%	23%	30%
Poz.o	53%	17%	31%
B.o	44%	26%	31%

Average profile	46%	24%	30%

**Table 7:** Frequency of breathing difficulties.

Symptom breathing difficulties	Age 6-7 (Ch1)	Age 13-14 (Ch2)	Age 20–44 (Ad)
Kat.o	18%	23%	19%
Zam.o	15%	14%	11%
Kr.o	21%	23%	17%
Wr.o	25%	24%	26%
L.o	19%	22%	16%
Gd.o	26%	30%	26%
Wa.o	32%	27%	26%
Poz.o	18%	19%	12%
B.o	18%	18%	18%

**Table 8:** Row profiles.

Symptom breathing difficulties	Age 6-7 (Ch1)	Age 13-14 (Ch2)	Age 20–44 (Ad)
Kat.o	30%	38%	32%
Zam.o	38%	35%	28%
Kr.o	34%	38%	28%
Wr.o	33%	32%	35%
L.o	33%	39%	28%
Gd.o	32%	37%	32%
Wa.o	38%	32%	31%
Poz.o	37%	39%	24%
B.o	33%	33%	33%

Average profile	34%	36%	30%

**Table 9:** Frequency of declared asthma.

Declared asthma	Age 6-7 (Ch1)	Age 13-14 (Ch2)	Age 20–44 (Ad)
Kat.a	5%	5%	4%
Zam.a	4%	4%	2%
Kr.a/L.a	6%	6%	5%
Wr.a	7%	10%	6%
Gd.a	3%	8%	5%
Wa.a	5%	8%	5%
Poz.a	5%	6%	4%
B.a	2%	4%	3%

**Table 10:** Row profiles.

Declared asthma	Age 6-7 (Ch1)	Age 13-14 (Ch2)	Age 20–44 (Ad)
Kat.a	36%	36%	29%
Zam.a	40%	40%	20%
Kr.a/L.a	35%	35%	29%
Wr.a	30%	43%	26%
Gd.a	19%	50%	31%
Wa.a	28%	44%	28%
Poz.a	33%	40%	27%
B.a	22%	44%	33%

Average profile	30%	42%	28%

**Table 11:** Percentage of declared asthma among respondents with “wheezing and whistling.”

Declared asthma	Age 6-7 (Ch1)	Age 13-14 (Ch2)	Age 20–44 (Ad)
Kat	21%	23%	14%
Zam	18%	32%	15%
Kr	17%	25%	29%
Wr	19%	38%	27%
L	22%	20%	22%
Gd	11%	31%	16%
Wa	11%	36%	18%
Poz	21%	43%	23%
B	6%	21%	13%

**Table 12:** Row profile.

Declared asthma	Age 6-7 (Ch1)	Age 13-14 (Ch2)	Age 20–44 (Ad)
Kat	37%	39%	24%
Zam	27%	49%	24%
Kr	24%	36%	40%
Wr	23%	45%	32%
L	35%	31%	35%
Gd	19%	54%	27%
Wa	17%	55%	27%
Poz	24%	50%	26%
B	16%	51%	33%

Average profile	25%	46%	30%
